# Postnatal development of the epididymis from birth until puberty of indigenous sheep (Barind-type) in Bangladesh

**DOI:** 10.5455/javar.2025.l972

**Published:** 2025-12-25

**Authors:** Mst. Aesha, Md. Sheikh Sadi, Md. Emtiaj Alam, Takashi Tanida, Md. Royhan Gofur

**Affiliations:** 1 Department of Veterinary and Animal Sciences, University of Rajshahi, Rajshahi, Bangladesh; 2 Department of Veterinary Anatomy, Graduate School of Veterinary Science, Osaka Metropolitan University, Izumisano, Osaka, Japan

**Keywords:** Epididymis, biometry, histomorphometry, Barind type indigenous sheep, postnatal development

## Abstract

**Objectives:**

The present study investigated the biometrical and histomorphometric changes in the epididymis of Barind-type indigenous sheep during postnatal development from birth to puberty.

**Materials and Methods:**

A total of 21 Barind-type indigenous ram lambs of varying postnatal ages—day 0, 1, and 2 weeks; and 1, 2.5, 5, and 7 months—were used in this study. Histomorphometric analysis was done using routine haematoxylin and eosin staining.

**Results:**

The Barind-type indigenous ram lamb epididymis followed a regionalized pattern of development, with the tail beginning development before other anatomical regions. The Barind-type indigenous ram lamb epididymal development was slow until 1 month of age, followed by a rapid period of epididymal development.The epididymal length and weight were more than 5 and 50 times greater, respectively, at puberty than at birth. The epithelial height and tubular diameter were expanded significantly (*p <* 0.05) from 1 month of age in all regions except in the tail at 7 months, where the epithelial height decreased dramatically (*p <* 0.05). Regarding regional differences, the largest epithelium height and tubular diameter were consistently found in the tail, and the smallest in the head, until 5 months. At 7 months, the reverse was observed in epithelium height. Epididymal regions were significantly (*p <* 0.05)different from one another at 5 and 7 months in terms of tubular diameter and epithelium height, respectively. Sperm first appeared in the tubular lumen by 7 months in the Barind-type indigenous ram epididymis.

**Conclusion:**

The biometric and histomorphometric alterations in the epididymal structure of Barind-type indigenous ram during postnatal development until puberty are recorded for the first time in this study.

## Introduction

Sheep, a globally distributed small ruminant, are crucial for meat, milk, and wool production, serving as a vital source of income for farmers with minimal investment and management [1]. In Bangladesh, the majority of sheep are nondescript indigenous types, lacking a specific breed designation [2]. However, these native sheep possess remarkable genetic qualities, including high prolificacy, early maturity, disease resistance, and adaptability to diverse environments [3].

The maturation of spermatozoa, which are necessary for fertilization, depends on the epididymis developing properly after birth. Testicular spermatozoa that are immature develop the ability to move and fertilize when they pass through the epididymis [4,5]. Disturbances in epididymal development or function can cause male infertility. The epididymis, at birth, is poorly developed and undergoes a complex development process during postnatal life, including elongation and coiling of the duct [6,7]. Initially, it contains many undifferentiated columnar epithelial cells and a few mitotic cells, which continue to divide. As the epididymis matures, these epithelial cells differentiate into principal cells and basal cells, ultimately leading to a functional organ capable of sperm maturation [5,8].

Thus, postnatal biometrical and histomorphometrical studies on the epididymis are important for understanding the epididymal developmental process, as well as the maturation and storage of spermatozoa. Recently, the details of postnatal testicular development in Black Bengal goats [9] and indigenous sheep [10] have been published; however, there has been no report published about the postnatal development of the epididymis of Barind-type indigenous sheep. Therefore, this study is designed to account for the changes in biometrical and histomorphological parameters of the epididymis of Barind-type indigenous sheep during postnatal development, providing useful data to understand the maturation of the epididymal duct as well as the maturation of spermatozoa in the Barind-type indigenous ram.

## Materials and Methods

### Ethical approval

All experimental procedures involving animals were conducted in accordance with the ethical guidelines established by the University of Rajshahi’s Animal Ethics Committee. The study was approved under Memo No. 293(13)/320/IAMEBBC/IBSc.; date: 18.08.2021.

### Experimental design including animals

The study was conducted using a total of 21 indigenous Barind-type sheep (*Ovis aries*) of varying postnatal ages: day 0, 1, 2 weeks, and 1, 2.5, 5, and 7 months. Ram lambs were reared with their dams under standard housing and feeding conditions. Guidelines established by the University of Rajshahi’s Ethical Committee were followed for conducting animal research (Memo No. 293(13)/320/IAMEBBC/IBSc). Ram lambs were surgically castrated under local anesthesia, and the epididymides were collected. After that, the left epididymides were preserved in a 10% formalin solution for histomorphometric analysis, whereas the right epididymides were used for biometric analysis.

***Biometric analysis***

The epididymis weight was measured (in gm) with an electric balance. Anatomically, the epididymis is divided into three regions: the head (caput or globus major), the body (corpus), and the tail (cauda or globus minor) [11]. The length and width of each region were measured (in cm) with the help of a vernier caliper.

### Histomorphometric analysis

Paraffin-embedded tissue sections (5 µm thick) of formalin-fixed left epididymis (from head, body, and tail regions) were cut using a sliding microtome (Thermo, Germany) and stained with routine hematoxylin and eosin stain according to Gofur et al. [9]. A compound microscope with magnifications of 10 and 40 was used to closely examine the stained slides of the developing epididymis. Using an oculometer (Erma, Japan) with a calibrated scale, the epithelial height as well as epididymal duct diameter of three distinct sections of the epididymis were measured (at 10×), and captured the photographs of these stained sections were captured using a photographic microscope system (Digital camera model: C-B5, OPTIKA, Italy fitted with a microscope, Model B-293PLi, OPTIKA, Italy).

### Statistical analysis

Values were shown as mean ± SE. Normality of the data was tested using the Shapiro-Wilk test. All variables were normally distributed (*p <* 0.05), so parametric analyses were applied. Parametric analyses of biometric and histomorphometric measurements among the epididymal regions and at various postnatal ages were performed using ANOVA followed by Tukey’s HSD post-hoc analysis, as described by Sadi and Gofur [10]. *p*-values of 0.05 or less were considered substantial differences.

## Results

### Biometry of postnatal developing epididymis of Barind type indigenous male lambs

**Figure 1.**
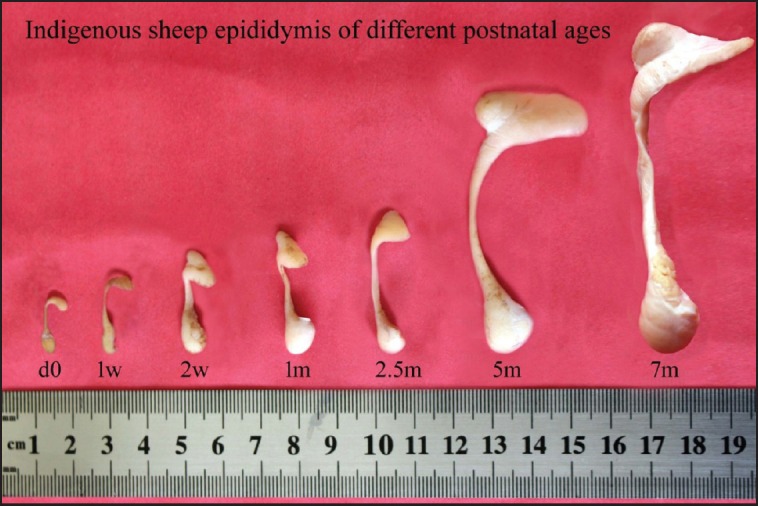
Gross images of the indigenous sheep epididymis at various stages of postnatal development. d0, day 0 or at birth; w, week; m, months.

Three parts or regions of the epididymis—head, body, and tail—were clearly identifiable in day-old lambs (Fig. 1). The typical figures of the postnatal growing epididymis of Barind-type indigenous male lambs of different age groups are presented in Figure 1, and their biometrical data (weight, length, and width) are shown in Table 1. The epididymal biometric values were increased (*p <* 0.05) with the progression of lamb age. The epididymal biometric values differed across various postnatal age groups. Up to around 1 month, the epididymal development was slow, as shown by insignificant biometrical values among age groups (d0 *vs.* 1 w *vs.* 2 w *vs.* 1 m), and then we observed a rapid epididymal development indicated by a significant difference (*p <* 0.05) in most of the biometrical parameters among age groups (1 m *vs.* 2.5 m *vs.* 5 m *vs.* 7 m) until puberty. The average epididymal length (head + body + tail) was more than 5 times greater at puberty (11.35 cm) than that at birth (2.04 cm), whereas the average weight at puberty (8.44 gm) was around 50 times more than the birth weight (0.17 gm) of lambs.

**Table 1.** Biometrical values (mean ± SE) of the epididymis of Barind type indigenous male lambs of various postnatal developing ages (*n* = 21).

Age	Weight (gm)	Head of epididymis		Body of epididymis		Tail of epididymis	
		Length (cm)	Width (cm)	Length (cm)	Width (cm)	Length (cm)	Width (cm)
d0	0.17 ± 0.01^a^	0.62 ± 0.03^a^	0.31 ± 0.01^a^	0.86 ± 0.03^a^	0.20 ± 0.01^a^	0.56 ± 0.02^a^	0.28 ± 0.01^a^
1 w	0.22 ± 0.01^a^	0.75 ± 0.04^ab^	0.41 ± 0.02^ab^	0.99 ± 0.02^ab^	0.21 ± 0.02^a^	0.64 ± 0.03^a^	0.34 ± 0.02^a^
2 w	0.35 ± 0.02^ab^	0.94 ± 0.04^ab^	0.50 ± 0.02^ab^	1.14± 0.03^ab^	0.26 ± 0.02^a^	0.76 ± 0.02^ab^	0.47 ± 0.02^ab^
1 m	0.52 ± 0.02^ab^	1.15± 0.06^b^	0.62 ± 0.03^bc^	1.30 ± 0.06^b^	0.32 ± 0.02^ab^	0.89 ± 0.05^b^	0.61 ± 0.04^b^
2.5 m	0.89 ± 0.04^b^	1.68 ± 0.07^c^	0.77 ± 0.04^c^	1.86 ± 0.08^c^	0.45 ± 0.03^b^	1.21 ± 0.06^c^	0.86 ± 0.06^c^
5 m	2.57 ± 0.17^c^	2.82 ± 0.10^d^	1.29 ± 0.08^d^	3.93 ± 0.10^d^	0.61 ± 0.05^c^	1.53 ± 0.04^d^	1.31 ± 0.06^d^
7 m	8.44 ± 0.28^d^	3.56 ± 0.17^e^	2.19 ± 0.10^e^	5.69 ± 0.15^e^	0.72 ± 0.06^d^	2.10 ± 0.08^e^	1.88 ± 0.06^e^

Note: Significant variations for postnatal ages are indicated by values in a column with distinct superscripts (*p <* 0.05). d0, day 0 or at birth; w, week; m, months.

### Characteristics of the epididymal tissue

**Figure 2.**
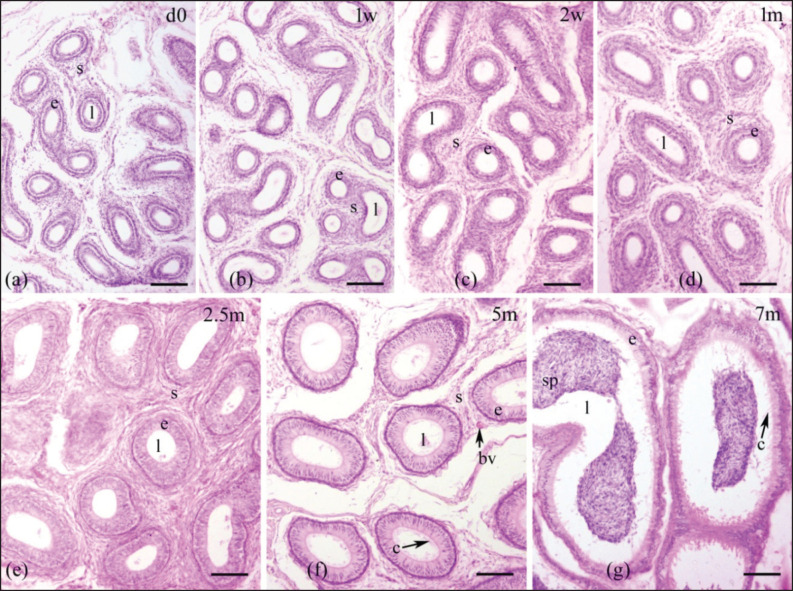
Histological images illustrating the epididymal ducts of the head region of the epididymis in indigenous sheep of various postnatal ages. The developing epididymis was lined by pseudostratified columnar epithelium. The epithelial height and tubular diameter were gradually increasing throughout the study period. Stereocilia were observed in the upper part of the epithelium on 5 and 7 months. Sperm were observed on 7 months. d, day; w, week; m, months; e, epithelium; s, stroma; l, lumen, c, cilia; bv, blood vessel; sp, spermatozoa; scale bar 100 μm.

**Figure 3.**
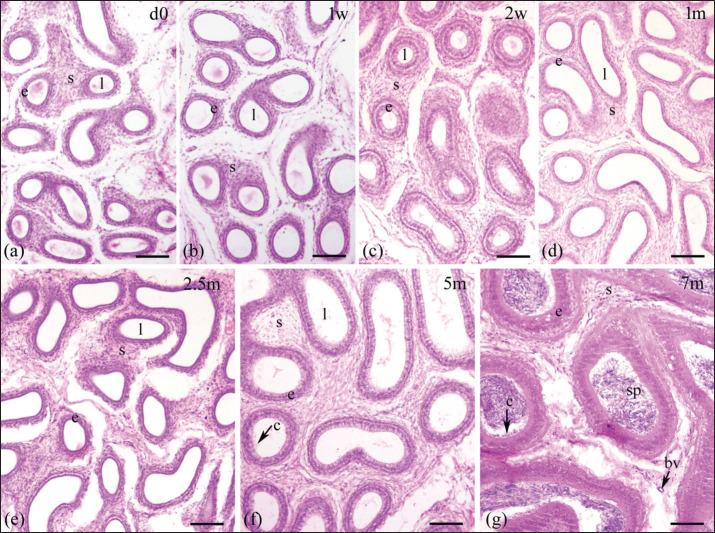
Histological images illustrating the epididymal ducts of the body region of the epididymis in indigenous sheep of various postnatal ages. The developing epididymis was lined by pseudostratified columnar epithelium. The epithelial height and tubular diameter were gradually increasing throughout the study period. Stereocilia were observed in the upper part of the epithelium on 5 and 7 months. Sperm were observed on 7 months. d, day; w, week; m, months; e, epithelium; s, stroma; l, lumen, c, cilia; bv, blood vessel; sp, spermatozoa; scale bar 100 μm.

**Figure 4.**
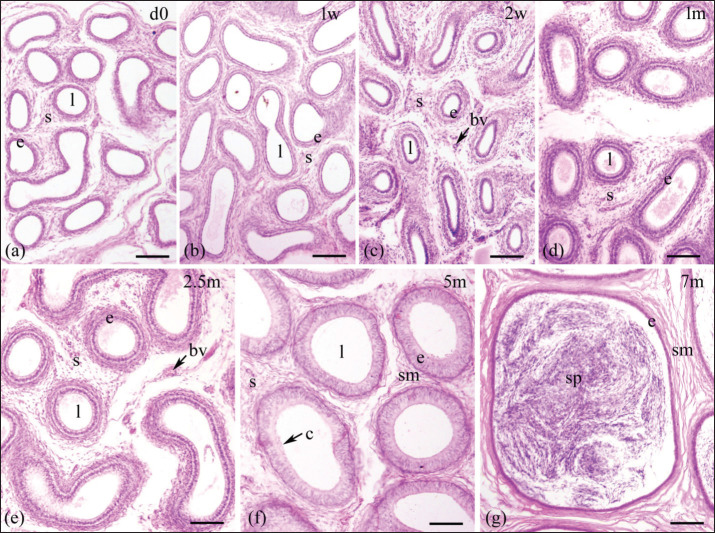
Histological images illustrating the epididymal ducts of the tail region of the epididymis in indigenous sheep of various postnatal ages. The developing epididymis was lined by pseudostratified columnar epithelium. The epithelial height and tubular diameter were gradually increasing throughout the study period except in tail on 7 months when the height of epithelium was drastically reduced. Stereocilia were observed in the upper part of the epithelium on 5 and 7 months. Sperm were observed on 7months. d, day; w, week; m, months; e, epithelium; s, stroma; l, lumen, c, cilia; bv, blood vessel; sm, smooth muscle; sp, spermatozoa; scale bar 100 μm.

**Figure 5.**
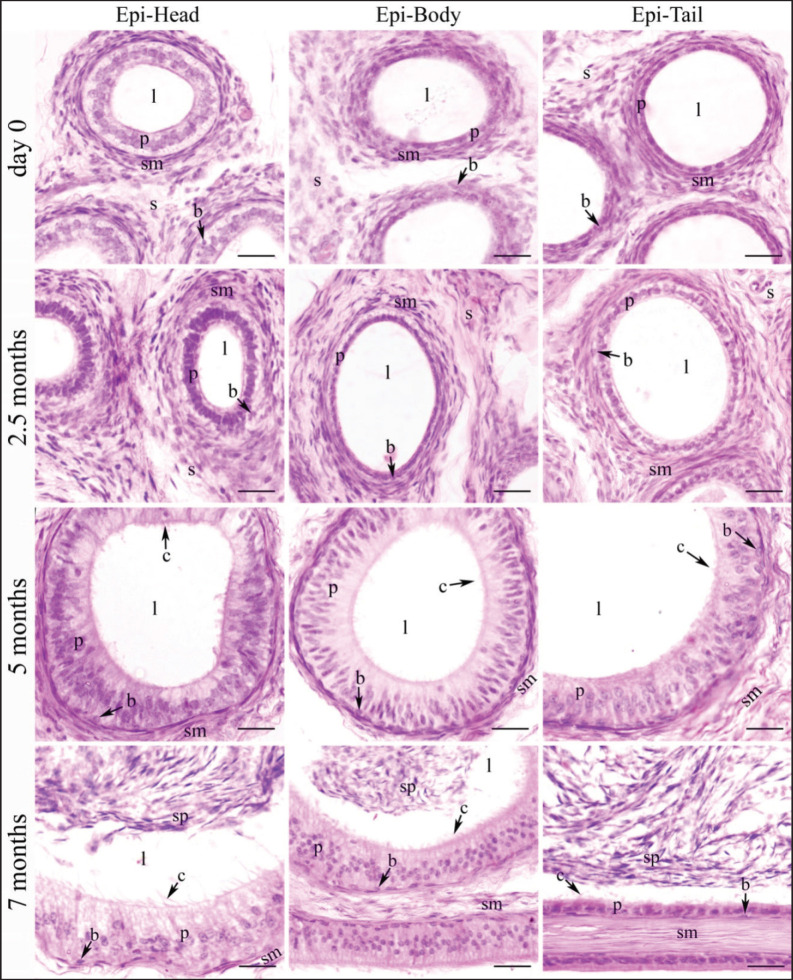
Histological images illustrating the epididymal ducts of different regions of indigenous sheep epididymis of various postnatal ages (at higher magnification). The developing epididymis was lined by pseudostratified columnar epithelium consisting with tall columnar principal cells and short cuboidal basal cells and encircled by some layers of smooth muscle. The epithelial height and tubular diameter were gradually increasing throughout the study period except in tail on 7 months when the height of epithelium was drastically reduced. Stereocilia were observed in all three regions at 5 and 7 months. Sperm were observed in all three regions at 7 months. Epi, epididymis; d, day; w, week; m, months; p, principal cells; b, basal cell; l, lumen; c, cilia; s, stroma; sm, smooth muscle; sp, spermatozoa; scale bar 20 μm.

**Figure 6.**
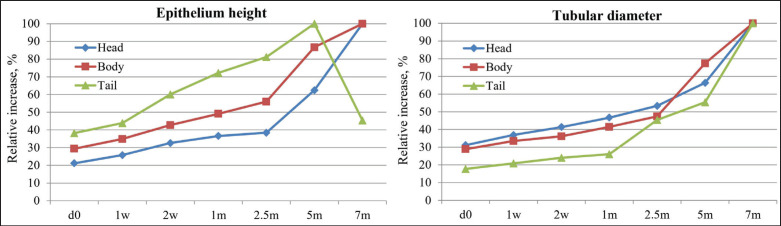
Relative increase of the epithelium height (left panel) and tubular diameter (right panel) at different postnatal developing ages in indigenous sheep. d, day; w, week; m, months. The data for epithelium height (left panel) show the percentage value at each age in relation to the highest value attained in each region (7 m for head and body, and 5 m for tail). Data are the percentage of values at each age relative to the value at 7 m of age for tubular diameter (right panel).

Epididymal ducts (ductus epididymis), which make up the epididymal parenchyma, are lined by pseudostratified columnar epithelium consisting of tall columnar principal cells and short cuboidal basal cells. This epithelial layer was scattered within a stroma and encircled by a few layers of smooth muscle (Figs. 2–5). The postnatal development of the epididymis in indigenous sheep involves changes in the height of the lining epithelium and the diameter of the epididymal ducts. Histomorphometric analysis reveals these changes with advancing age. The lining epithelium transitions from ciliated low columnar to ciliated pseudostratified columnar epithelium, starting in the tail and progressing to the body and head. Additionally, the epithelial height and tubular diameter increase from birth to late pre-puberty (Table 2, Fig. 6).

**Table 2.** Epithelium height and tubular diameter (mean ± SE) in different epididymal anatomical regions in Barind type indigenous male lambs, from birth to puberty (*n* = 21).

Age	Epithelium height (µm)			Tubular diameter (µm)		
	Head	Body	Tail	Head	Body	Tail
d0	^a^11.8 ± 0.92^y^	^a^12.8 ± 0.97^yz^	^a^16.2 ± 1.36^z^	^a^76 ± 2.92^y^	^a^80 ± 3.54^y^	^a^93 ± 2.55^z^
1w	^ab^14.4 ± 1.12^y^	^ab^15.2 ± 0.97^yz^	^ab^18.6 ± 1.17^z^	^ab^90 ± 3.54^y^	^ab^96 ± 3.32^y^	^ab^110 ± 3.54^z^
2w	^abc^18.2 ± 1.46^y^	^abc^18.6 ± 1.17^y^	^bc^25.4 ± 2.48^z^	^ab^101 ± 2.92^y^	^ab^104 ± 3.67^y^	^ab^126 ± 4.30^z^
1m	^bc^20.4 ± 0.98^y^	^bc^21.4 ± 1.17^y^	^cd^30.6 ± 1.3^z^	^bc^114 ± 2.92^y^	^bc^119 ± 5.10^y^	^b^145 ± 7.58^z^
2.5m	^c^22.2 ± 1.28^y^	^c^24.4 ± 1.69^y^	^d^34.4 ± 1.69^z^	^c^130 ± 3.54^y^	^c^136 ± 4.30^y^	^c^239 ± 7.48^z^
5m	^d^34.8 ± 2.60^y^	^d^37.8 ± 2.35^yz^	^e^44.4 ± 2.21^z^	^d^162 ± 7.18^x^	^d^222 ± 8.60^y^	^d^292 ± 10.68^z^
7m	^e^55.8 ± 2.29^z^	^d^43.6 ± 2.44^y^	^ab^19.2 ± 1.02^x^	^e^244 ± 12.19^x^	^d^287 ± 10.44^y^	^e^527 ± 23.54^z^

Values with different superscript alphabets (^a-e^) within the same column signify a significant age difference (*p <* 0.05), and different superscript alphabets (^x, y, z^) within the row signify a significant epididymal regional difference (*p <* 0.05). d, day; w, week; m, months.

### Histomorphometry of the postnatal developing epididymis of native sheep

#### Epididymal epithelium

An increasing trend in epithelial height was observed in all three regions throughout the study period except in the tail at 7 months, when the height of the epithelium was drastically reduced (Table 2, Fig. 6). In the head and body, the epithelial height did not significantly change until 2 weeks from birth, then significantly (*p <* 0.05) increased until 7 months (Table 2); in the tail, a noticeable upward tendency was observed from 2 weeks to 5 months, then drastically (*p* < 0.05) decreased at 7 months of age (Table 2, Fig. 6). Comparing among epididymal regions, two distinct phases were included: from birth to 5 months, the epithelial height was the highest in the tail and the lowest in the head (*p <* 0.05), but at 7 months the situation reversed: the lowest value was noticed in the tail and the greatest in the head (*p <* 0.05). At 7 months of postnatal age, each epididymal region was significantly (*p <* 0.05) different from others in terms of epithelial height and ductal diameter (Table 2, Figs. 2–5). Stereocilia were observed from 5 months of postnatal age in all three regions of the Barind-type indigenous sheep epididymis (Figs. 2–5).

#### Tubular diameter

A relative increasing trend in tubular diameter was observed in all three regions of the epididymis throughout the study period (Table 2, Figs. 5,6). The tubular diameter did not change significantly from birth until 2 weeks, then increased significantly (*p* < 0.05) until 7 months in all three regions (Table 2). Regarding epididymal regional difference, the greatest diameter of the epididymal ducts was typically observed in the tail region, while the smallest diameter was found in the head (*p <* 0.05). The histomorphometric values of the head and body were similar, except at 5- and 7-month postnatal ages, when all regions differed from one another (Table 2). The appearance of sperm in all three regions of the epididymis’ tubular lumen was observed by 7 months of age in Barind-type indigenous ram lambs (Figs. 2–5), which strongly suggests that they have either reached or are very close to reaching puberty.

## Discussion

Understanding the development of the epididymis is crucial for comprehending male reproductive health and fertility. The epididymis plays a vital role in sperm maturation, storage, and transport, and its proper development is essential for these functions [4]. A thorough understanding of this development can also help identify and address abnormalities that may lead to infertility or other reproductive issues. Increasing sperm concentration in semen, a crucial component of male fertility, requires sperm concentration in the epididymis [4]. The size of the epididymis in animals is important because it directly relates to sperm storage, maturation, and overall reproductive function. A larger epididymis generally indicates a greater capacity for sperm storage, which can be important for species with extended mating periods or high sperm output [12].

The biometrical values of the Barind-type indigenous lamb epididymis increase greatly with age, consistent with prior research [13,14] in other small ruminants. This indicates a developmental growth trend in the epididymis as the animal ages. When lambs reached puberty, their average epididymal weight was approximately 50 times greater than when they were born. In Barbados Blackbellyram lambs, the weight of the epididymis was 0.47 gm at birth and increased around 27-fold at 21 weeks of age [14]. However, the epididymal weight may vary depending on the individual, season of birth, diet, breed, and management. The lamb epididymal average length was 5 times more at puberty than at birth. Moreover, the body had the largest length among the three parts of the epididymis, followed by the head and the shorter tail. The long length of the head and body may take more time for the sperm to travel; by this time, the sperm become mature and motile, as the proximal parts of the epididymis (head and body) are involved in the sperm maturation process [15,16].

Epididymal postnatal development followed a similar pattern to the testicular postnatal development in Barind-type indigenous sheep. Earlier, Sadi and Gofur [10] studied the testicular postnatal development in Barind-type indigenous sheep and observed a steady and gradual testicular development with age. A similar observation was also reported for Blackbelly ram lambs [17]. During postnatal development, an increase in testicular interstitial tissue, particularly the number and steroidogenic capacity of Leydig cells [18], leads to higher testosterone levels and, consequently, greater stimulation of epididymis development. This is because the development and function of the epididymis are reliant on sufficient androgen contribution, with testosterone being the primary androgen [19,20].

The epididymal ducts (ductus epididymides), which make up the epididymal parenchyma, are lined by pseudostratified columnar epithelium consisting of tall columnar principal cells and short cuboidal basal cells. This epithelial layer was scattered within a stroma and encircled by a few layers of smooth muscle. The stereocilia were observed from 5 months of postnatal age in all three regions in Barind-type indigenous ram lambs. The histological architecture of the epididymis of Barind-type indigenous ram lambs was like that of other animals [21–25].

In the epididymis of Barind-type indigenous ram lambs, a significant difference in epithelial height and epididymal duct diameter was observed between different postnatal age groups and regional variations. Epithelial height and tubular diameter of the epididymis increased with age, showing the greatest values in the tail and the lowest in the head. At 7 months, epithelial height in the tail markedly declined, and all three regions differed significantly. A similar trend of age and regional difference in ductal diameter and epithelial height is described in Barbados Blackbelly ram lambs [14] and in Ouled Djellal lambs [26] during the postnatal development. Meng et al. [27] also observed the regional histological variations in the ductus epididymidis of the Congjiang Xiang pig. Pilutin et al. [28] and Arrighi et al. [29] also stated a comparable pattern in ductal diameter and epithelial height in rat and Mediterranean Italian buffalo epididymis, respectively, despite the fact that the values differ.

Gofur et al. [24] and Zayed et al. [30] characterized the differences between the head and tail of the epididymis in pubertal indigenous buffalo bulls and in one-humped camels, respectively, noting that the tail, which is the storage area for mature sperm, has a low epithelium and a wide lumen filled with spermatozoa. In contrast, the head of the epididymis, where sperm are initially processed and begin their maturation, features a tall epithelium with long stereocilia and a lumen containing mass collections of spermatozoa. The findings above and the observations obtained in the current investigation on Barind-type indigenous ram lambs are nearly identical.

The current study, along with prior research in various species [24,28,30], confirms a progressive reduction in the height of the epithelial cells lining the epididymal duct as one moves from the head (caput) to the tail (cauda) of the epididymis. This decrease in epithelial height is observed particularly near the onset of puberty. The progressive reduction in epithelial height within the epididymal duct, from the head to the tail, can facilitate sperm’s journey through the duct. This reduction in epithelial height likely creates a smoother, more streamlined flow within the duct, aiding in sperm transport. Normally, the proximal regions of the epididymis (head and body) are primarily involved in the maturation of spermatozoa, while the tail of the epididymis serves as the main storage site for mature spermatozoa [15,16]. However, a high epithelium (tall columnar principal cells) in the head could indicate that the head region’s epithelium has a greater potential for absorption, like the high (tall columnar) epithelium in the proximal part of the nephron, where the most absorption occurs [31]. James et al. [4] describe that the head (caput) of the epididymis absorbs more than 90% of the fluids entering the epididymal duct in bulls. This fluid reabsorption is a crucial function of the epididymis, enabling the concentration of sperm and preparing them for storage and transport. On the other hand, the enlarged tubular lumen of the tail of the epididymis allows for a larger volume of mature sperm to be held, and also helps to maintain sperm viability and motility until ejaculation [32].

According to the present study, sperm were first observed in the epididymal lumen of all three regions of the epididymis in Barind-type indigenous sheep by 7 months, which strongly suggests that these sheep have either reached or are very close to reaching puberty by this age. This is because sperm production and maturation in the epididymis are key indicators of reproductive maturity [33]. Ghezel ram lambs reach puberty by 8 months after birth, according to Nazari-Zenouz et al. [34]. Belkhiri et al. [26] reported that at 8 months of age, a large number of spermatozoa were accumulated in the cauda, corpus, and caput epididymis lumens, and mature spermatozoa were greater in number in the cauda epididymis during puberty than in the corpus or caput epididymis in Ouled Djellal lambs. Moreover, the age of puberty or sexual maturity of Barind-type indigenous ram lambs may vary depending on the nutrition, season of birth, breed, and management [35].

## Conclusion

This is the first detailed description of the morphometry of the Barind-type indigenous sheep epididymis during postnatal development. In Barind-type indigenous ram lambs, epididymal development was slow until 1 month, then accelerated, with the tail maturing earliest. By 7 months, sperm appeared in all regions, indicating puberty, though the tail showed a sharp decline in epithelial height. The development of the epididymis may serve as a reliable indicator of reproductive maturity and fertility and could potentially be used to select male ram lambs with higher breeding potential. The present study’s results will aid in identifying developmental abnormalities and pathologic or toxicological alterations in the epididymis of the Barind-type indigenous ram, and the fundamental morphological analyses conducted in this study may be crucial for future research on reproductive biology and breeding.

## List of abbreviations

n, number; gm, grams; cm, centimeter; μm, micrometer; d, day; w, week; m, month; SE, standard error.
